# A biological Bayesian network for prediction of adverse outcome in a population of acutely ill patients triaged in the Emergency Department

**DOI:** 10.1186/1757-7241-21-S2-A11

**Published:** 2013-09-09

**Authors:** Charlotte Barfod, Lars Hyldborg Lundstrøm, Kai Henrik Wiborg Lange, Kristen Barfod

**Affiliations:** 1 Deparment of Anaesthesiology and Intensive Care Medicine, Nordsjællands Hospital Hillerød; 2 National Food Institute, Technical University of Denmark, Copenhagen

## Background

We know from previous studies that increasing age, abnormal vital signs and abnormal acid-base status are strongly associated with in-hospital mortality in unselected patients admitted acutely to hospital. A model including this information will make us able to explore associations and predict the risk for future patients. Our aim was to describe a Bayesian model for prediction of adverse outcome in the acute ill adult patient admitted to hospital, based on already existing data from the ‘Acute Admission Database’.

## Methods

The model is a static Bayesian network, i.e. a stochastic model where all interdependence is described by conditional probabilities. The net consists of nodes representing variables and pointed arrows of influence. The probabilities connected to the nodes and arrows are conditional probabilities showing how the state of a variable influences the probability distribution for the states of another variable. We based the model on already existing data from the ‘Acute Admission Database’ and imported data from 6279 patients consecutively admitted to Hillerød Hospital through the Emergency Department into the Bayesian net program, Netica “3.7” © Norsys Software Corp. We included the risk factors identified in this cohort in previous studies as nodes, and represented the known associations with directed arrows.

## Results

We tested the use of the model by simulating the path of an acutely ill patient: a male patient, 70 years old and presenting with vomiting blood. By using this evidence in the nodes of relevance, we could assess the most probable distribution of the other nodes, including the outcome of interest. We simulated that more data became available for instance vitals signs and triage categories. This new evidence changed the nodes and finally we entered information about a venous blood gas, which changed the probability distribution of the outcome measures as more evidence was gained.

## Conclusion

By using already existing data, we were able to build a Bayesian network, which can be used to estimate the risk of adverse outcome and serve as a decision support system in assessing future patients admitted acutely to hospital.

